# Diagnosis and management of Sandifer syndrome in children with intractable neurological symptoms

**DOI:** 10.1007/s00431-019-03567-6

**Published:** 2020-01-11

**Authors:** Irina Mindlina

**Affiliations:** School of Clinical Medicine, University of Cambridge, Hills Road, Cambridge, CB2 0QQ UK

**Keywords:** Sandifer syndrome, Gastro-oesophageal reflux disease, Misdiagnosis, Intractable neurological symptoms

## Abstract

Sandifer syndrome is a rare complication of gastro-oesophageal reflux disease (GERD) when a patient presents with extraoesophageal symptoms, typically neurological. The aim of this study was to review the existing literature and describe a typical presentation and most appropriate investigations and management for the Sandifer syndrome. A comprehensive literature search was performed via PubMed, Cochrane Library and NHS Evidence databases. Twenty-seven cases and observational studies were identified. The literature demonstrates that presenting symptoms of Sandifer’s may include any combination of abnormal movements and/or positioning of head, neck, trunk and upper limbs, seizure-like episodes, ocular symptoms, irritability, developmental and growth delay and iron-deficiency anaemia. A 24-h oesophageal pH monitoring was positive in all the cases of Sandifer’s where it was performed, while upper GI endoscopy ± biopsy and barium swallow were diagnostic only in a subset of cases. Successful treatment of the underlying gastro-oesophageal pathology led to a complete or near-complete resolution of the neurological symptoms in all of the cases.

*Conclusion*: It is evident from the literature that many patients with Sandifer syndrome were originally misdiagnosed with various neuropsychiatric diagnoses that led to unnecessary testing and ineffective medications with significant side effects. Earlier diagnosis of Sandifer’s would have allowed to avoid them.

**Table:** 

**What is Known:** • *Sandifer syndrome is a rare complication of gastro-oesophageal reflux disease (GERD) when a patient presents with extraoesophageal symptoms, typically neurological.* • *It may be difficult to recognise due to its non-specific presentation and lack of gastrointestinal symptoms.*	
**What is New:** • *Based on the review of 44 clinical cases of suspected Sandifer syndrome, the clinical picture was clarified: the presenting symptoms of Sandifer’s may include any combination of abnormal movements and/or positioning of head, neck, trunk and upper limbs, seizure-like episodes, ocular symptoms, irritability, developmental and growth delay and iron-deficiency anaemia.* • *Successful treatment of the underlying gastro-oesophageal pathology led to a complete or near-complete resolution of the neurological symptoms in all of the reviewed cases.*

**What is Known:**

• *Sandifer syndrome is a rare complication of gastro-oesophageal reflux disease (GERD) when a patient presents with extraoesophageal symptoms, typically neurological.*

• *It may be difficult to recognise due to its non-specific presentation and lack of gastrointestinal symptoms.*

**What is New:**

• *Based on the review of 44 clinical cases of suspected Sandifer syndrome, the clinical picture was clarified: the presenting symptoms of Sandifer’s may include any combination of abnormal movements and/or positioning of head, neck, trunk and upper limbs, seizure-like episodes, ocular symptoms, irritability, developmental and growth delay and iron-deficiency anaemia.*

• *Successful treatment of the underlying gastro-oesophageal pathology led to a complete or near-complete resolution of the neurological symptoms in all of the reviewed cases.*

## Introduction

Sandifer syndrome is defined as a rare complication of gastro-oesophageal reflux disease (GERD) when a patient presents with extraoesophageal symptoms, typically neurological [18]. These symptoms may be as severe as mimicking epileptic seizures or convulsions [23]. Although its pathophysiology is not completely understood, one possible explanation is that neurological manifestations are the consequence of vagal reflex with the reflex center in nucleus tractus solitarii [3]. The main difficulty with accurately diagnosing this clinical presentation is that often the overt gastro-intestinal symptoms, such as abdominal pain, vomiting or indigestion, are either absent, or the patient is too young to be able to communicate them. Thus, there is nothing to point the clinician to the direction of GI investigations, and as a result, the vast majority of patients with Sandifer syndrome are originally misdiagnosed with a neurological or a musculoskeletal disorder. This may lead to a range of unnecessary investigations, such as EEG, MRI and electromyographic studies, all of which come back normal. Moreover, this may result in the administration of unnecessary medications, such as anti-epileptic agents, which may have significant negative side effects. The aim of this study is to review the existing literature and describe a typical presentation and most appropriate investigations and management for the Sandifer syndrome, so that it can be considered early on in the differential diagnosis for children with intractable neurological symptoms.

## Methodology

The literature search strategy included conducting a systematic review via Cochrane Library, PubMed and NHS Evidence databases. The search terms and the outcomes are listed below:

- Cochrane library: ‘Sandifer* syndrome’
  - 1 controlled trial, not relevant
- PubMed search: ‘Sandifer* syndrome’
  - 86 results in the English language, 27 were relevant
  - The following publications were excluded:
    - Case studies covering only adults
    - Correspondence that did not include specific patient cases
- NHS Evidence: ‘Sandifer* syndrome’
  - 8 results, 1 was relevant (NICE guideline)

Overall, 27 case reports and observational studies were available for analysis, covering 44 clinical cases in total.

## Results

The detailed findings of the systematic review are provided in Table 1.

**Table 1:** Data collected from the literature review

Ref. no.	Reference	Study group size	Key results				Comments: prior misdiagnosis (e.g. neurological)?
			Extraoesophageal symptoms	Diagnostic investigation	Definitive treatment	Outcome: complete resolution of symptoms?	
[1]	Bamji et al. (2015)	2	Abnormal body posturing, irritability	None, empirical treatment	Dietary	Yes	
[20]	Nalbantoglu et al. (2013)	1	Abnormal posturing and movement of head and neck; ocular deviation	Oesophageal pH monitoring, oesophageal biopsy	Dietary	Yes	
[30]	Tokuhara et al. (2008)	1	Growth retardation, abnormal neck movement, anaemia, hypoproteinaemia	Upper GI endoscopy with biopsy, 24-h pH monitoring	Surgical	Yes	
[15]	Kostakis et al. (2008)	1	Abnormal head posturing	n/a^1^	n/a^1^	n/a^1^	
[16]	Lehwald et al. (2007)	1	Abnormal posturing and movement of head and neck	Upper GI endoscopy	Surgical	Near complete	Yes
[9]	Firat et al. (2007)	1	Abnormal movements of head and neck, motor and speech delay	Oesophageal pH monitoring, upper GI fluoroscopy	Surgical	Yes	Yes
[13]	Kabakuş, Kurt (2006)	4	Abnormal posturing and movements of head and neck, ocular deviation, irritability, growth retardation, anaemia	Gastro- oesophageal scintigraphies	Dietary and lifestyle, pharmacological	Near complete	Yes
[5]	Corrado et al. (2006)	1	Abnormal posturing and movement of head, neck and trunk	24-h gastro-oesophageal pH monitoring	Pharmacological	Yes	
[10]	Frankel et al. (2006)	1	Abnormal head posturing, irritability	Oesophageal pHmetry, surface electromyography, split-screen videography	Surgical	Yes	Yes
[7]	Demir et al. (2001)	1	Abnormal positioning and movements of neck, hand tremor, vomiting, stridor	Barium oesophagogram	Pharmacological	n/a^1^	
[6]	de Ybarrondo, Mazur (2000)	1	Cerebral palsy, severe developmental delay, asthma	n/a^1^	n/a^1^	n/a^1^	
[4]	Corrado et al. (2000)	1	Abnormal movements of head, neck, trunk	24-h pH oesophageal monitoring	Dietary	Yes	
[29]	Tekou et al. (1997)	1	Abnormal posturing of head and neck	n/a^1^	Surgical	Yes	
[2]	Cardi et al. (1996)	1	n/a^1^	Real-time ultrasonography	n/a^1^	n/a^1^	
[8]	Deskin (1995)	1	Abnormal neck posturing, irritability, cough, hoarseness	Barium swallow	Surgical	Yes	
[11]	Gorrotxategi et al. (1995)	8	Abnormal neck posturing	Barium swallow, 24-h pH-metering, manometry, endoscopy, biopsy	Surgical (3 patients), pharmacological (5 patients)	Significant improvement	
[26]	Senocak et al. (1993)	1	Abnormal neck posturing	n/a^1^	Surgical	Yes	
[28]	Sommer (1993)	13	Developmental delay, abnormal behaviour, hoarse growling cry (all patients had Brachmann-de Lange syndrome)	3 patients–barium swallow, 10 patients–pH probe monitoring of upper GI system, esophagoscopy, endoscopy	Pharmacological and dietary (5 patients), surgical (8 patients)	Significant improvement	
[25]	Puntis et al. (1989)	1	Abnormal posturing and movements of head, neck, trunk, anaemia	Barium swallow, oesophageal pH monitoring, upper GI endoscopy and biopsy	Surgical	Yes	
[17]	Mandel et al. (1989)	3	Abnormal posturing and movements of head and trunk, weight loss	12-h lower oesophageal pH monitoring	Pharmacological	Yes	Yes
[21]	Nanayakkara, Paton (1985)	3	Abnormal posturing and movements of head, neck and trunk	Barium study	Pharmacological, dietary	Yes	
[12]	Hadari et al. (1984)	1	Abnormal posturing and movements of body and limbs, seizure-like episodes, hypotonia, developmental delay	Barium study	Pharmacological	Yes	Yes
[21]	Nanayakkara, Paton (1985)	3	Abnormal movements of neck and trunk, irritability	n/a^1^	Pharmacological	Yes	Yes
[12]	Hadari et al. (1984)	1	Abnormal posturing and movements of head and neck	n/a^1^	Surgical	Yes	
[14]	Keren et al. (1983)	1	Abnormal posturing and movement of head and trunk	Barium study	n/a^1^	n/a^1^	
[27]	Smallpiece, Deverall (1982)	1	Irritability, abnormal posturing and movements of head, neck, trunk, growth delay	Barium swallow	Surgical	Yes	
[32]	Werlin et al. (1980)	5	Abnormal body posturing	n/a^1^	n/a^1^	n/a^1^	
[19]	Murphy, Gellis (1977)	2	Abnormal neck posturing	XR studies	Pharmacological	Yes	
[24]	O’Donnell, Howard (1971)	1	Abnormal head and neck posturing, strabismus, anaemia	n/a^1^	n/a^1^	n/a^1^	Yes

^1^Information not available

The literature demonstrates that the presenting symptoms of Sandifer syndrome may include any combination of abnormal movements and/or positioning of head, neck, trunk and upper limbs, seizure-like episodes, ocular symptoms, irritability, developmental and growth delay and iron-deficiency anaemia. It is evident from the literature that many of the patients were originally misdiagnosed with various neuropsychiatric diagnoses that led to unnecessary testing and ineffective medications that may have caused significant side effects. Earlier diagnosis of Sandifer’s would have allowed to avoid them.

As Sandifer syndrome is caused by gastro-oesophageal reflux, its investigations and management should be consistent with those of GERD. In terms of diagnostic procedures, 24-h oesophageal pH monitoring was positive in all the cases of Sandifer’s where it was performed, while upper GI endoscopy ± biopsy and barium swallow were diagnostic only in a subset of cases.

A range of treatment options were applied in the reviewed literature, including dietary changes (cow’s milk exclusion, amino-acid-based formula), pharmacological management (alginates, proton pump inhibitors (PPIs)), enteral tube feeding, and surgical approach, when conservative management was ineffective (Nissen fundoplication is usually curative). The pharmacological treatment was often sufficient on its own to achieve the resolution of symptoms; however, further escalation of management was required in the cases of advanced disease. These treatment options are consistent with the 2015 NICE guideline on management of GERD in children and young people [22].

Successful treatment of the underlying gastro-oesophageal pathology led to a complete or near-complete resolution of the neurological symptoms in all of the reviewed cases.

## Discussion

1. Sandifer syndrome may be difficult to recognise due to its non-specific presentation; however, it is an important differential diagnosis to consider in children with neurological symptoms that remain unexplained by neurological investigations.
2. When Sandifer syndrome is suspected, 24-h oesophageal pH monitoring is usually diagnostic; however, an empirical trial of pharmacological management (e.g., prescribing a PPI) is also appropriate without prior invasive investigation [31].
3. Once diagnosed, it can be successfully managed by treating the underlying GERD/hiatus hernia which typically leads to a complete resolution of all associated symptoms.
4. In the majority of patients, pharmacological management is sufficient for the resolution of symptoms. Other treatment options include dietary modifications, enteral tube feeding, and surgical management.
5. The choice of a management plan in each case depends on the severity and duration of the underlying condition, as well as individual responsiveness to treatment.

## Abbreviations

EEG: Electroencephalography
GERD: Gastro-oesophageal reflux disease
GI: Gastro-intestinal
MRI: Magnetic resonance imaging
NICE: National Institute of Clinical Excellence (UK)
NHS: National Health Service (UK)
PPI: Proton pump inhibitor

## References

[1] Bamji N, Berezin S, Bostwick H, Medow MS (2015). Treatment of Sandifer syndrome with an amino-acid-based formula. AJP Rep.

[2] Cardi E, Corrado G, Cavaliere M, Capocaccia P, Matrunola M, Rea P, Pacchiarotti C (1996). Delayed gastric emptying in an infant with Sandifer syndrome. Ital J Gastroenterol.

[3] Cerimagic D, Ivkic G, Bilic E (2008). Neuroanatomical basis of Sandifer’s syndrome: a new vagal reflex?. Med Hypotheses.

[4] Corrado G, Cavaliere M, D'Eufemia P, Pelliccia A, Celli M, Porcelli M, Giardini O, Cardi E (2000). Sandifer’s syndrome in a breast-fed infant. Am J Perinatol.

[5] Corrado G, Fossati C, Turchetti A, Pacchiarotti C, Nardelli F, D'Eufemia P (2006). Irritable oesophagus: a new cause of Sandifer’s syndrome. Acta Paediatr.

[6] de Ybarrondo L, Mazur JL (2000). Sandifer’s syndrome in a child with asthma and cerebral palsy. S Med J.

[7] Demir E, Saka E, Aysun S (2001). A case of Sandifer’s syndrome with hand tremor. Turk J Pediatr.

[8] Deskin RW (1995). Sandifer syndrome: a cause of torticollis in infancy. Int J Pediatr Otorhinolaryngol.

[9] Firat AK, Karakas HM, Firat Y, Yakinci C (2007). Unusual symptom of intestinal malrotation: episodic cervical dystonia due to Sandifer syndrome. Pediatr Int.

[10] Frankel EA, Shalaby TM, Orenstein SR (2006). Sandifer syndrome posturing: relation to abdominal wall contractions, gastroesophageal reflux, and fundoplication. Dig Dis Sci.

[11] Gorrotxategi P, Reguilon MJ, Arana J, Gaztañaga R, Elorza C, de la Iglesia E, Barriola M (1995). Gastroesophageal reflux in association with the Sandifer syndrome. Eur J Pediatr Surg.

[12] Hadari A, Azizi E, Lernau O, Nissan S (1984). Sandifer’s syndrome--a rare complication of hiatal hernia. A case report. Z Kinderchir.

[13] Kabakuş N, Kurt A (2006). Sandifer syndrome: a continuing problem of misdiagnosis. Pediatr Int.

[14] Keren G, Frand M, Jonas A, Avigad I, Rotem Y (1983). Sandifer’s syndrome following reverse gastric tube operation (Gavriliu’s operation). J Pediatr Surg.

[15] Kostakis A, Manjunatha NP, Kumar A, Moreland ES (2008). Abnormal head posture in a patient with normal ocular motility: Sandifer syndrome. J Pediatr Ophthalmol Strabismus.

[16] Lehwald N, Krausch M, Franke C, Assmann B, Adam R, Knoefel WT (2007). Sandifer syndrome--a multidisciplinary diagnostic and therapeutic challenge. Eur J Pediatr Surg.

[17] Mandel H, Tirosh E, Berant M (1989). Sandifer syndrome reconsidered. Acta Paediatr Scand.

[18] Moore DM, Rizzolo D (2018). Sandifer syndrome. JAAPA.

[19] Murphy WJ, Gellis SS (1977). Torticollis with hiatus hernia in infancy. Sandifer syndrome. Am J Dis Child.

[20] Nalbantoglu B, Metin DM, Nalbantoglu A (2013). Sandifer's syndrome: a misdiagnosed and mysterious disorder. Iran J Pediatr.

[21] Nanayakkara CS, Paton JY (1985). Sandifer syndrome: an overlooked diagnosis?. Dev Med Child Neurol.

[22] National Institute for Health and Care Excellence (2015) Gastro-oesophageal reflux disease in children and young people: diagnosis and management [NG1]. https://www.nice.org.uk/guidance/ng1. Accessed 12 Oct 2018 31944641

[23] Nuysink J, van Haastert IC, Takken T, Helders PJ (2008). Symptomatic asymmetry in the first six months of life: differential diagnosis. Eur J Pediatr.

[24] O'Donnell JJ, Howard RO (1971). Torticollis associated with hiatus hernia (Sandifer’s syndrome). Am J Ophthalmol.

[25] Puntis JW, Smith HL, Buick RG, Booth IW (1989). Effect of dystonic movements on oesophageal peristalsis in Sandifer’s syndrome. Arch Dis Child.

[26] Senocak ME, Arda IS, Büyükpamukçu N (1993). Torticollis with hiatus hernia in children. Sandifer syndrome. Turk J Pediatr.

[27] Smallpiece CJ, Deverall PB (1982). Sandifer’s syndrome: a new cause. Thorax.

[28] Sommer A (1993). Occurrence of the Sandifer complex in the Brachmann-de Lange syndrome. Am J Med Genet.

[29] Tekou H, Akue B, Senah KC, Etey K, Dagnra PC (1997). Sandifer’s syndrome--a report of one case. West Afr J Med.

[30] Tokuhara D, Yamano T, Okano Y (2008). A case of Sandifer’s syndrome: significance in the differential diagnosis of growth retardation. J Paediatr Child Health.

[31] Vandenplas Y, Ashkenazi A, Belli D, Boige N, Bouquet J, Cadranel S, Cezard JP, Cucchiara S, Dupont C, Geboes K (1993). A proposition for the diagnosis and treatment of gastro-oesophageal reflux disease in children: a report from a working group on gastro-oesophageal reflux disease. Eur J Pediatr.

[32] Werlin SL, D'Souza BJ, Hogan WJ, Dodds WJ, Arndorfer RC (1980). Sandifer syndrome: an unappreciated clinical entity. Dev Med Child Neurol.

